# Macroautophagy-Mediated Degradation of Whole Nuclei in the Filamentous Fungus *Aspergillus oryzae*

**DOI:** 10.1371/journal.pone.0015650

**Published:** 2010-12-20

**Authors:** Jun-ya Shoji, Takashi Kikuma, Manabu Arioka, Katsuhiko Kitamoto

**Affiliations:** Department of Biotechnology, The University of Tokyo, Tokyo, Japan; Iowa State University, United States of America

## Abstract

Filamentous fungi consist of continuum of multinucleate cells called hyphae, and proliferate by means of hyphal tip growth. Accordingly, research interest has been focusing on hyphal tip cells, but little is known about basal cells in colony interior that do not directly contribute to proliferation. Here, we show that autophagy mediates degradation of basal cell components in the filamentous fungus *Aspergillus oryzae*. In basal cells, enhanced green fluorescent protein (EGFP)-labeled peroxisomes, mitochondria, and even nuclei were taken up into vacuoles in an autophagy-dependent manner. During this process, crescents of autophagosome precursors matured into ring-like autophagosomes to encircle apparently whole nuclei. The ring-like autophagosomes then disappeared, followed by dispersal of the nuclear material throughout the vacuoles, suggesting the autophagy-mediated degradation of whole nuclei. We also demonstrated that colony growth in a nutrient-depleted medium was significantly inhibited in the absence of functional autophagy. This is a first report describing autophagy-mediated degradation of whole nuclei, as well as suggesting a novel strategy of filamentous fungi to degrade components of existing hyphae for use as nutrients to support mycelial growth in order to counteract starvation.

## Introduction

Autophagy is the process whereby cytoplasmic compounds and organelles are sequestered into vacuoles for degradation and recycling [1]. For instance, in yeast autophagy is known to play a key role in degrading cytoplasmic organelles such as mitochondria [2] and peroxisomes [3], and recycling them as nutrients in order for cells to survive starvation. There are two distinct but related mechanisms for autophagy, termed micro-, and macroautophagy. In microautophagy, organelles are recruited at the cytoplasmic surface of vacuolar membrane invaginations, which are then pinched-off to incorporate organelles into the vacuolar lumen [1]. Macroautophagy begins by formation of crescent-like precursors of autophagosomes that then mature into ring-shaped double membrane autophagosomes to encapsulate organelles. The outer membrane of the autophagosomes then fuses with the vacuolar membrane to release the inner membrane and its contents into the vacuoles [1]. Degradation products such as amino acids are then translocated to the cytoplasm via vacuolar membrane transporters to be reused [4].

Although the molecular mechanism of autophagy has been extensively studied in yeast cells [1], there is also accumulating evidence revealing importance of autophagy in development and growth of filamentous fungi. In the rice blast fungus *Magnaporthe oryzae*, autophagy has an important role in conidial programmed cell death and appressorium formation that are prerequisites for its pathogenicity. [5], [6], [7], [8]. In other fungi, autophagy is known to be important for differentiation, such as aerial hypha formation, and growth in a starvation condition [9], [10], [11].

Filamentous fungi typically consist of continuum of multinucleate cells called hyphae. Hyphal growth at the tips is the major way in which they proliferate, and generally basal cells in old hyphae do not directly contribute to the proliferation. In our previous report, we described that in basal cells of the filamentous fungus *Aspergillus oryzae*, vacuoles occupy nearly the entire volume of the cells [12]. As the increase of the vacuolar volume in these cells should have been accompanied by a decrease of the cytoplasmic volume, we speculated that autophagy takes place in these cells to degrade and recycle cytoplasmic and organellar components. In this report, we used several fungal strains in which cytoplasmic organelles are labeled with enhanced green fluorescent protein (EGFP) to test their uptake by vacuoles via autophagy. Unexpectedly, we found that whole nuclei, which have never been reported as substrates of autophagy, were taken up via macroautophagy and degraded within vacuoles. We also demonstrated that autophagy is essential for efficient growth when the availability of extracellular nutrients is low. Our results suggest new strategy of filamentous fungi to reuse nuclei as nutrient pools to assist the tip growth through the cellular nutrient recycling system.

## Results

To test the hypothesis that cytoplasmic organelles in basal cells of a filamentous fungus are degraded through autophagy, we used several *A. oryzae* strains in which established markers of organelles are labeled by EGFP fusion proteins. The marker proteins included peroxisome-targeting signal 1 (PTS1) as a peroxisomal marker [13], citrate synthase as a mitochondrial marker [14], and histone H2B as a nuclear marker [15]. We observed EGFP fluorescence in young growing hyphae at one day, and in basal cells at two days to investigate the autophagic uptake of these organellar markers in old hyphae.

**Figure 1:**
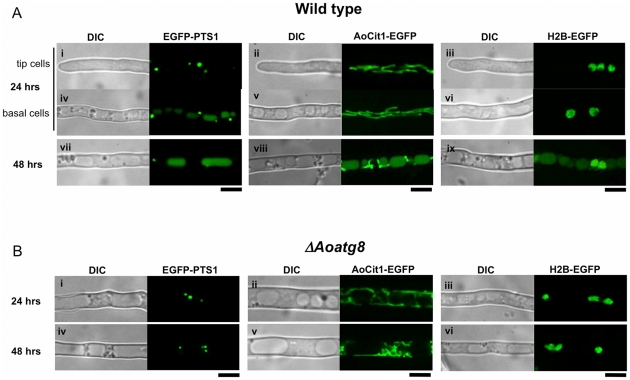
Autophagy-dependent uptake of EGFP labeling peroxisomes, mitochondria, and nuclei in basal cells of *A. oryzae*. Subcellular distribution of EGFP labeling peroxisomes (Ai, iv, vii, Bi, iv), mitochondria (Aii, v, viii, Bii, v), and nuclei (Aiii, vi, ix, Biii, vi) in either wild-type (A) or an *Aoatg8*-deleted background (B). Cultures were incubated at 30°C for either 24 hrs (Ai–vi, Bi–iii) or 48 hrs (Avii–ix, Biv–vi). Bars represent 5 µm.

In apical cells from 1-day cultures, EGFP visualized punctate peroxisomes, filamentous mitochondria, and round nuclei (Figure 1Ai–iii), respectively, consistent with previous reports [13], [14], [15]. However, we noticed that a portion of the EGFP fluorescence derived from peroxisomes, the representative substrates for autophagy [3], was already detected within vacuoles in basal cells at this stage (Figure 1Aiv). EGFP fluorescence derived from mitochondria or nuclei was not detected within vacuoles at this incubation period (Figure 1Av, vi). The accumulation of EGFP fluorescence from EGFP-PTS1 in vacuoles was more pronounced in 2-day-old cultures in which tip growth had arrested and most cells had basal cell-like appearance. Predominant EGFP fluorescence was detected in vacuoles in the EGFP-PTS1-expressing strain (Figure 1Avii). Remarkably, EGFP fluorescence was also found in vacuoles of strains expressing AoCit1-EGFP (Figure 1Aviii) and H2B-EGFP (Figure 1Aix) in 2-day-old cultures. These results demonstrated that components of peroxisomes, mitochondria, and nuclei are taken up by vacuoles in basal cells. In the absence of functional *Aoatg8*, whose product is essential for autophagy [10], no EGFP fluorescence from any of the fusion proteins was observed in vacuoles even in 2-day-old (Figure 1Bi–vi) or later cultures. These results suggested that autophagy mediates the uptake of peroxisomes, mitochondria, and nuclei in basal cells of *A. oryzae* to recycle their components as nutrients.

It is well established in budding yeast that peroxisomes [3] and mitochondria [2] are subjected to autophagy-mediated degradation upon suitable environmental stimuli. Furthermore, in budding yeast a portion of its nucleus is taken up by microautophagy and degraded within vacuoles upon starvation [16]. However, this process only degrades pre-ribosomes and a part of the nuclear envelope. By contrast, because we used EGFP-fused histone H2B as a nuclear marker, our results suggested the occurrence of autophagy-mediated degradation of whole nuclei, including chromatin structures. As *A. oryzae* cells are multinucleate, the cells might be able to degrade and recycle nuclear composition as nutrients without causing cessation of their cellular activities. This prompted us to investigate further the mechanism by which EGFP fused to histone H2B is translocated into vacuoles.

There are two possible explanations for how nucleus-derived EGFP-H2B is incorporated into vacuoles. One possibility is that EGFP is dispersed throughout the cytoplasm after degradation of nuclei in the cytoplasm, and then it is taken up into vacuoles along with the cytosol by non-specific autophagy. The other explanation is that intact nuclei could be directly subjected to autophagic uptake by vacuoles. If the former were the case, we would expect to observe the similar number of nuclei between wild-type and the *Aoatg8*-deleted strains, while cytoplasmic EGFP fluorescence would be more pronounced in the latter strain. However, the average number of nuclei in 3-day-old cultures of the wild-type strain was significantly smaller than that in the *Aoatg8*-deleted strain (4.0±2.4 vs. 7.6±2.2 per 50 µm-long hyphae, average ± S. D., n = 100, p<0.001), suggesting that the *Aoatg8*-mediated autophagic pathway is involved in the regulation of the number of nuclei. There was no significant difference of hyphal diameter between wild-type and *Aoatg8*-deleted strains. Although nuclei in the *Aoatg8*-deleted strain were sometimes misshapen compared to those of the wild-type strain (Figure S1), we did not observe cytoplasmically scattered EGFP fluorescence in either strains (e.g., see Figure 1). Therefore, the accumulation of nucleus-derived EGFP in vacuoles was not due to degradation of nuclei within the cytoplasm and the subsequent uptake of EGFP by non-specific autophagy.

**Figure 2:**
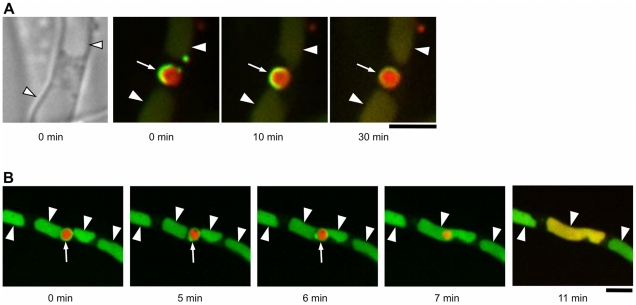
Macroautophagy-mediated uptake of nuclei by vacuoles. A series of superimposed images of EGFP-AoAtg8 and H2B-mDsRed is shown. (A) A crescent of an autophagosomal precursor (green, arrow) matured into a ring-like autophagosome to encircle an apparently intact nucleus (red). Arrowheads point to vacuoles. (B) A ring-like autophagosome (green, arrow) surrounding an apparently intact nucleus (red) disappeared (7 min), followed by dispersal of H2B-fused mDsRed throughout a vacuole. Arrowheads point to vacuoles. Bars represent 5 µm.

We next observed the behavior of nuclei in 2-day-old cultures. Although our previous study suggested occurrence of microautophagy in basal cells [12], we found no evidence for microautophagy-mediated uptake of nuclei. We then visualized autophagosomal structures, which mediate the uptake of macroautophagy substrates [1], using EGFP-fused AoAtg8 [10]. In 2-day-old cultures, ring-like EGFP fluorescence was frequently observed (Figure S2A). The appearance of these structures was very similar to that of autophagosomes in other systems [1], except that they were much larger (1–2 µm in diameter). As these ring-like structures were dependent on *Aoatg4* that is crucial for functional autophagy (Kikuma et al., in prep.), we concluded that they are large autophagosomes. Surprisingly, when nuclei were simultaneously visualized by mDsRed-fused histone H2B, every large autophagosome was observed to contain one, or sometimes two, apparently intact nuclei (Figure S2A, B). Time-lapse observation revealed that crescent-like precursors of autophagosomes eventually matured into ring-like autophagosomes to encircle nuclei (Figure 2A). When ring-like autophagosomes were further observed, EGFP fluorescence representing autophagosomes suddenly disappeared, followed by dispersal of H2B-mDsRed fluorescence throughout vacuoles (Figure 2B, Movie S1), indicating that the nuclei were incorporated in vacuoles and degraded. To further confirm the autophagic degradation of nuclei, we generated a strain lacking *Aoatg15*, an *A. oryzae* homologue of *ATG15* which encodes a vacuolar lipase required for degradation of autophagic vesicles [17]. When EGFP-AoAtg8 and H2B-mDsRed were simultaneously expressed in the *ΔAoatg15* background, mDsRed fluorescence representing nuclei was found as shrunk structures in vacuole-like compartments where EGFP-AoAtg8, which most likely represents autophagic bodies, accumulated (Figure S3). However, mDsRed fluorescence dispersed throughout vacuoles was never observed, suggesting that the nuclei were incorporated into vacuoles but were not degraded because of the loss of the lipase necessary to degrade autophagosomal membrane. These results imply that whole nuclei, and not only a part of them, are substrates of autophagy, and suggest their recycling as nutrients.

**Figure 3:**
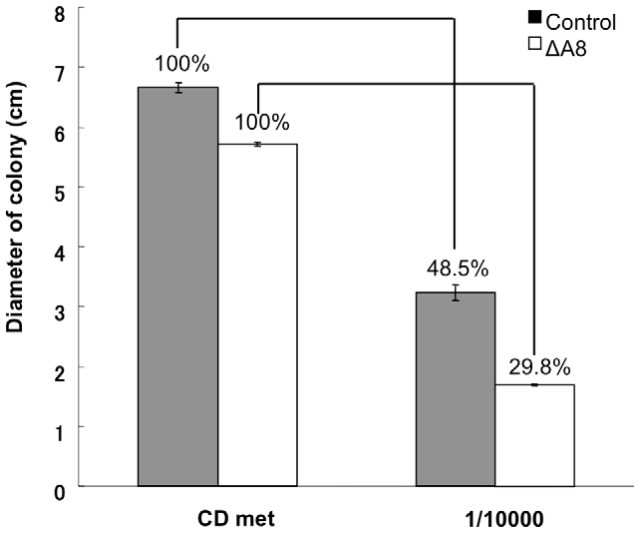
Autophagy supporting the mycelial growth. Wild-type and *ΔAoatg8* strains were inoculated and grown on either the CD medium with 0.15% methionine (nutrient-supplied) or its 10^4^ times dilution (nutrient-depleted) for seven days. The colony diameters of each strain at the seventh day are shown. Relative diameters of each strain to the nutrient-supplied medium are shown as percentages. Error bars represent S. D. n = 3.

The results so far indicated the occurrence of autophagy-mediated degradation of cytoplasmic organelles in basal cells of a filamentous fungus. Considering that autophagy is the well-defined intracellular recycling system, its degradation products are most likely to be reused as nutrients. Because the basal cells of filamentous fungi do not generally grow and their cellular activity is believed to be low, it is reasonable to speculate that degradation products from autophagy is somehow transported to hyphal tips to support the tip growth. As the fungus can also use extracellular nutrients to support its growth, autophagy-mediated recycling of basal cell components might become important when there are only limited nutrient sources available in the environment. To investigate if autophagy is important for hyphal growth in nutrient-depleted condition, we recorded colony diameters on agar plates of the wild-type and *ΔAoatg8* strains over seven days. The colony diameter after seven days of incubation was reduced by 52% in the wild-type strain grown in the nutrient-depleted medium, compared to the standard nutrient-supplied medium (Figure 3). The reduction was more drastic in the *ΔAoatg8* strain, and the colony diameter in the nutrient-depleted condition was only about 30% of that in the standard medium. The same experiment using the strains in which nuclei and autophagosomes were labeled confirmed that autophagic degradation of nuclei takes place in basal cells in these conditions (Figure S4). These results demonstrated that autophagy in basal cells is important for proliferation when starved, and suggests the recycling of basal cell components including nuclei as nutrients to support tip growth.

## Discussion

Our results demonstrated that basal cells of *A. oryzae* undergo global degradation of cytoplasmic organelles including nuclei, mitochondria, and peroxisomes by autophagy. The macroautophagy-mediated degradation of whole nuclei is of particular interest as it has never been reported in any organisms. Even though microautophagy-mediated degradation of nuclear components has been reported in budding yeast, this process only degrades a part of nuclei such as pre-ribosomes and nuclear envelopes [16]. Mammalian cells degrade nuclear components by macroautophagy, but again this only degrades a part of nuclei such as damaged DNA and proteins, and apparently it first requires translocation of the nuclear components into the cytoplasm which is followed by their macroautophagic degradation [18]. Thus, the key feature of macroautophagy-mediated degradation of nuclei we report here is that large autophagosomal structures encircle and mediate degradation of apparently whole nuclei. Since virtually all large autophagosomes contained one or more nuclei, these large autophagosomes are likely to be specifically dedicated to degradation of nuclei. Therefore, it seems the autophagic degradation of nuclei is a specific process, which may be regulated separately from autophagic degradation of other organelles. However, even though the autophagic degradation of peroxisomes initiated earlier than that of other organelles, it appears autophagy of the three organelles simultaneously takes place in basal cells of 1–2 days cultures. Thus, we can not exclude the possibility that the internal/external signals stimulating the autophagy of these organelles are identical.

Degradation of a whole nucleus would lead to eternal loss of its activity and genetic information therein. However, since filamentous fungi such as *A. oryzae* possesses multinucleate hyphae, degradation of one or a few nuclei would not cause the immediate cell death, and cells should still be able to translocate the degradation products to appropriate sites in the mycelium, most likely to hyphal tips where proliferation takes place. In accordance with this idea, both circumstantial [19] and experimental [11] evidence has suggested that autophagy supports proliferation of filamentous fungi by nutrient recycling. Furthermore, we showed that autophagy is in fact important for mycelial growth when nutrient availability is limited. Thus, autophagic degradation of basal cell components may provide a unique means to support hyphal growth by degrading cells that do not directly contribute to proliferation and using them as nutrient storage. This might be a key strategy of filamentous fungi living in the natural environment where nutrients are heterogeneously distributed. In such a condition, filamentous fungi colonize a nutrient source while hyphae which are not in contact with nutrients eventually regress [20]. These hyphae may not just die of starvation; rather, they might be undergoing active cell death by degrading their cellular components via autophagy. The resultant low-molecular-weight compounds, such as amino acids and polyphosphates, might be recycled to hyphal tips via the motile tubular vacuole systems that have been implicated in intra- and intercellular transport of nutrients [19], [21], [22] to serve as nutrients. The autophagy-mediated degradation of nuclear components might be especially important as nuclei contain abundant phosphorus and nitrogen, two major nutrients that are the most growth-limiting factors in the ecosystem for fungi [23]. Thus, the important future question to be addressed is whether degradation products derived from autophagic degradation of basal cell organelles are actually translocated to hyphal tips and used as nutrients, and whether autophagic degradation of nuclei has particular importance.

Although this report is the first to describe macroautophagy-mediated uptake of whole nuclei, this process might be a common feature among multinucleate organisms. For example, autophagy is accompanied by degeneration of nuclei during the onset of appressorium formation in the rice blast fungus *Magnaporthe oryzae* [5]. During this process, one nucleus moves to a forming appressorium, concomitant with translocation of other nuclei to the conidium, followed by their autophagy-dependent disappearance. In the ciliate *Tetrahymena thermophila*, macronuclei co-localize with compartments that are positive for acid phosphatase activity and then disappear, suggesting the occurrence of autophagic degradation of the macronucleus [24]. It might be possible that even mammalian cells recycle their nuclei in specific circumstances, considering that certain cell types are multinucleate [25], and that certain anucleate cells sustain their cellular activity [26]. Further analyses in these and other organisms will provide insights into how nuclei support the cellular physiology, in addition to their well-established role as the genetic information center.

## Materials and Methods

### Plasmid and strain construction

Plasmids and strains for visualization of nuclei (H2B-EGFP, [6]), peroxisomes (EGFP-PTS1, [4]), mitochondria (EGFP-AoCit1 [5]), and autophagosomal structures (EGFP-AoAtg8, [8]) were described previously. Essentially, H2B-EGFP and EGFP-AoAtg8 are under the regulation of their respective native promoters, whereas EGFP-PTS1 and EGFP-AoCit1 are expressed under the control of *A. oryzae amyB* promoter. All four fusion proteins were expressed ectopically.

A plasmid for expression of H2B-mDsRed fusion protein was constructed through the Multisite Gateway Cloning system (Invitrogen, [5]). *h2b* promoter and *h2b* ORF were amplified by PCR and introduced into 5′ and center entry clones, respectively. The inserted sequences were then confirmed by ABI PRISM™ 310NT Genetic Analyzer (Life Technologies Japan, Tokyo, Japan). These two entry clones were assembled with a 3′ entry clone containing *egfp* and a destination vector containing the *sC* marker [5] through a recombination reaction. The resultant plasmid was introduced into the *A. oryzae* NS4 strain using *sC* as the selective marker, yielding the strain HHDR. The plasmid encoding EGFP-AoAtg8 [8] was then introduced into this strain, resulting in HREA strain in which nuclei and autophagosomal structures are labeled with mDsRed and EGFP, respectively.

The *Aoatg8* deletion strain was described previously [8]. For deletion of *Aoatg4* or *Aoatg15*, knockout cassettes consisting of the 5′ flanking regions, the *adeA* marker, and 3′ flanking regions were prepared using the Multisite Gateway cloning system, and the NS4 strain was transformed using these cassettes. Recombination at the desired loci resulting in the deletion of respective genes was confirmed by Southern blotting using ECL direct nucleic acid labeling and detection systems (GE Healthcare Japan, Tokyo, Japan).

### Fungal strains and culture condition

*Aspergillus oryzae* strains used in this study are listed in Table S1. For visualization of peroxisomes, EGFP was fused to a peroxisome-targeting signal SRL [4]. Cultures were prepared in the Czapek-Dox minimal medium (0.3% NaNO3, 0.2% KCl, 0.1% KH2PO4, 0.05% MgSO4•7H2O, 0.002% FeSO4•7H2O, 2% glucose, pH 5.5) in glass base dishes (IWAKI, Tokyo, Japan). As a nutrient-depleted medium, the CD medium diluted 10^4^ times was used.

### Microscopy

Microscopy was performed using an IX71 inverted microscope (Olympus, Tokyo, Japan) equipped with 100x and 40x Neofluor objective lenses (1.30 numerical aperture), 488 nm (Furukawa Electric) and 561 nm (Melles Griot) semiconductor lasers, GFP, DsRed, and DualView filters (Nippon Roper, Tokyo, Japan), a CSU22 confocal scanning system (Yokogawa Electronics, Tokyo, Japan), and an Andor iXon cooled digital CCD camera (Andor Technology PLC, Belfast, Ireland). Images were analyzed with Andor iQ software (Andor Technology PLC).

## Supporting Information

Table S1 **Strains used in this study.**
(TIF)

Figure S1 **Shapes of nuclei visualized with EGFP-fused histone H2B in wild-type (A) and** ***ΔAoatg8*** **(B) strains.** Bar represents 5 µm.
(TIF)

Figure S2 **EGFP-AoAtg8-visualized autophagosomal structures that encircle mDsRed-H2B-visualized nuclei.**
A. Autophagosomal structures that encircle nuclei. A crescent-like autophagosomal precursor (arrow), ring-like autophagosomes (large arrowhead), and dot-like structures that might represent pre-autophagosomal structures (narrow arrowheads) are seen. B. An autophagosomal structure that encircles two nuclei. Bars represent 5 µm.
(TIF)

Figure S3 **Defective degradation of nuclei in the** ***ΔAoatg15*** **background.**
The *ΔAoatg15* strain was obtained through homologous recombination, and verified by Southern analysis. The *ΔAoatg15* strain simultaneously expressing EGFP-AoAtg8 and H2B-mDsRed was grown for 2 days and subjected to microscopy. The arrowhead points a nucleus which appears shrunk in a vacuole-like compartment where EGFP-AoAtg8, most likely representing autophagic bodies, accumulates. Note that there is no dispersed red fluorescence in vacuole-like compartments which are labeled by EGFP. The bar represents 5 µm.
(TIF)

Figure S4 **Macroautophagy-mediated degradation of a nucleus in a basal cell of an agar culture.**
The HREA strain simultaneously expressing EGFP-AoAtg8 and H2B-DsRed was inoculated on a 1000x diluted CD agar medium in a glass-base dish, and grown for two days at 30°C. A hypha in the colony center was pictured. The scale bar represents 5 µm.
(TIF)

Movie S1 **Degradation of an apparently intact nucleus in vacuoles.**
The red fluorescence (H2B-mDsRed) represents a nucleus and the green fluorescence (EGFP-AoAtg8) indicates an autophagosomal structure and vacuolar lumen. The movie was generated by pictures shown in Figure 2.
(MOV)
