# Supplementary figures and images for: Macroautophagy-Mediated Degradation of Whole Nuclei in the Filamentous Fungus Aspergillus oryzae
Source: PLoS One. 2010 Dec 20;5(12):e15650. doi: 10.1371/journal.pone.0015650 (PMC3004950; doi:10.1371/journal.pone.0015650)

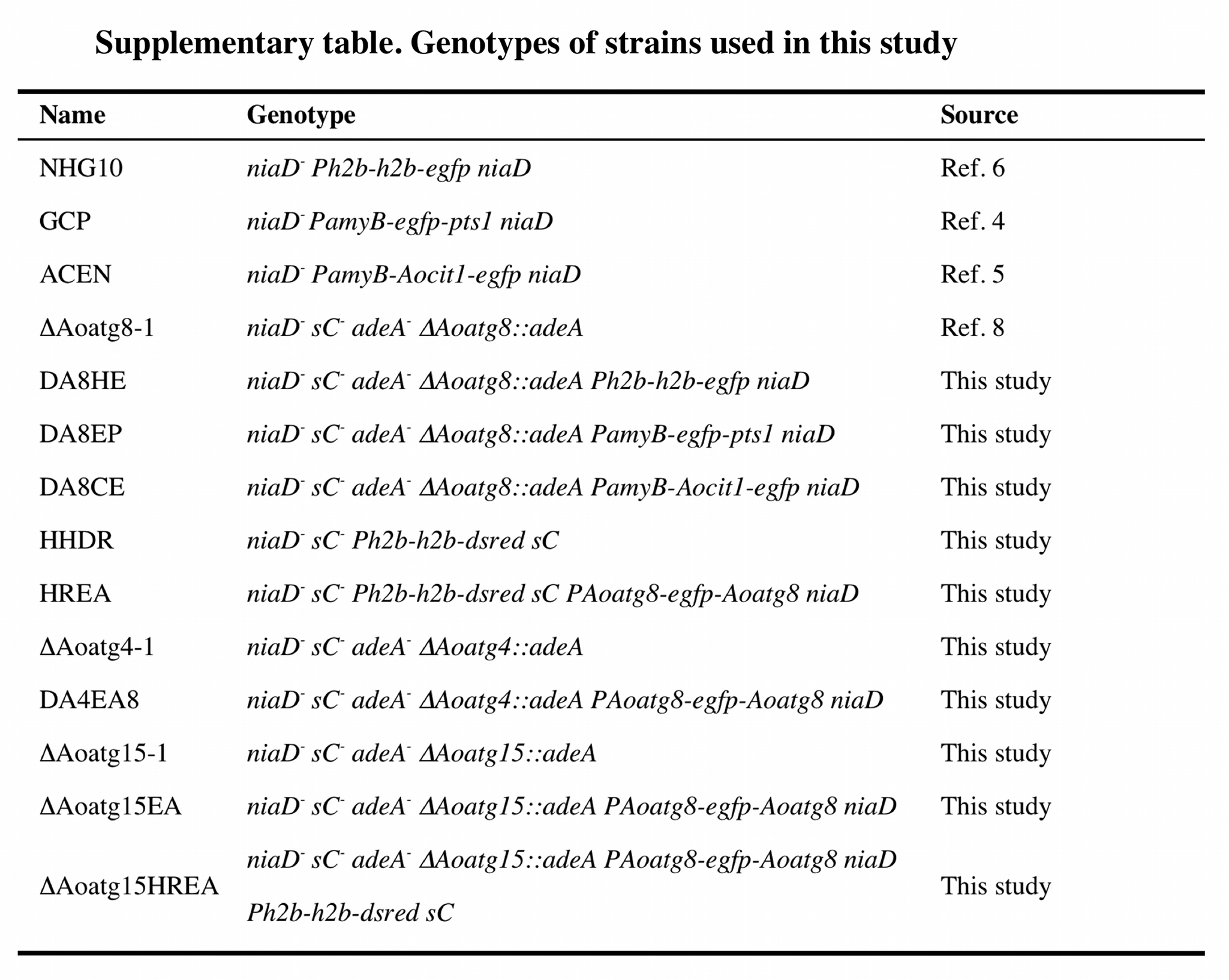

Supplement: Table S1 — Strains used in this study. (TIF) [file pone.0015650.s001.tif]

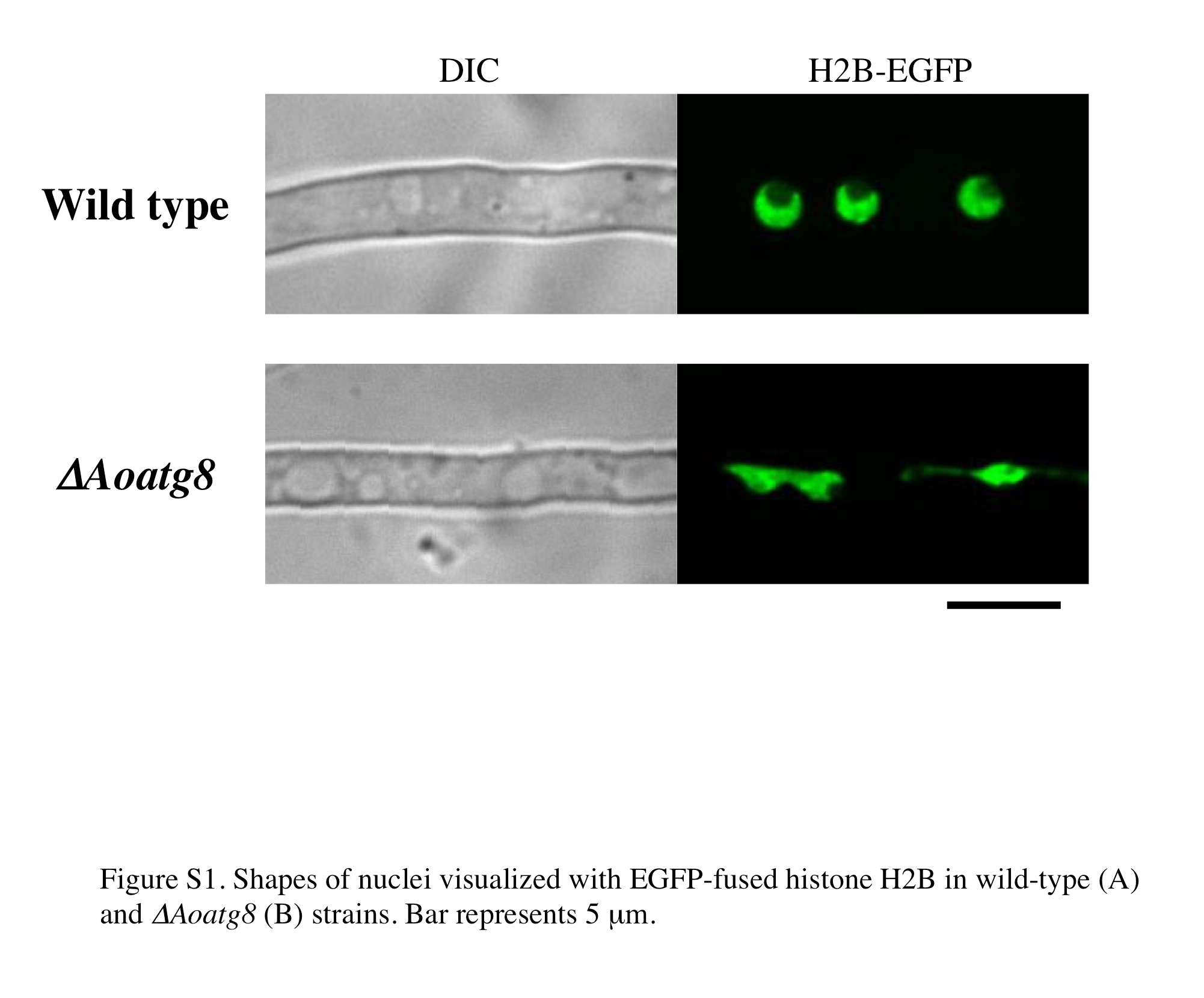

Supplement: Figure S1 — Shapes of nuclei visualized with EGFP-fused histone H2B in wild-type (A) and ΔAoatg8 (B) strains. Bar represents 5 µm. (TIF) [file pone.0015650.s002.tif]

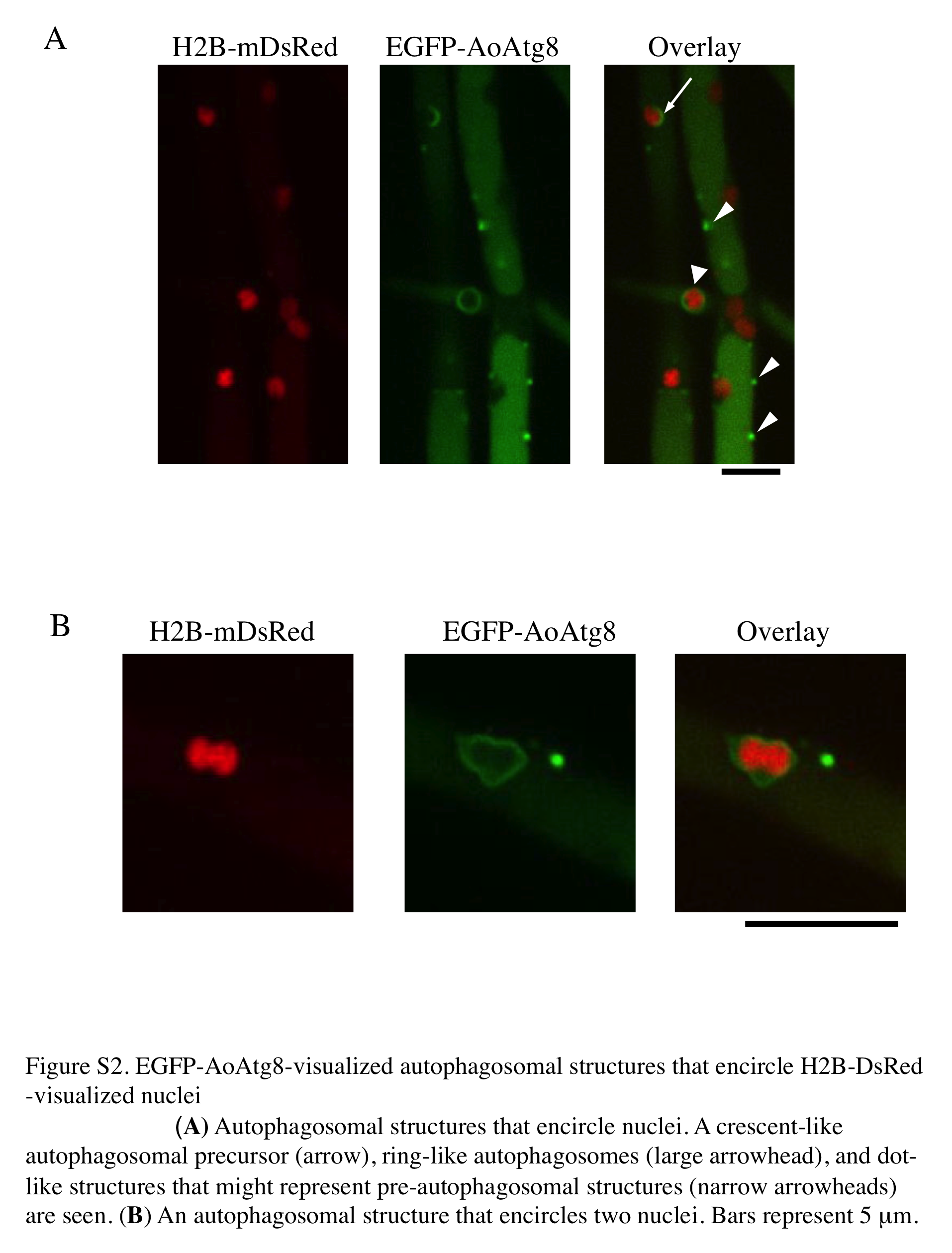

Supplement: Figure S2 — EGFP-AoAtg8-visualized autophagosomal structures that encircle mDsRed-H2B-visualized nuclei. A. Autophagosomal structures that encircle nuclei. A crescent-like autophagosomal precursor (arrow), ring-like autophagosomes (large arrowhead), and dot-like structures that might represent pre-autophagosomal structures (narrow arrowheads) are seen. B. An autophagosomal structure that encircles two nuclei. Bars represent 5 µm. (TIF) [file pone.0015650.s003.tif]

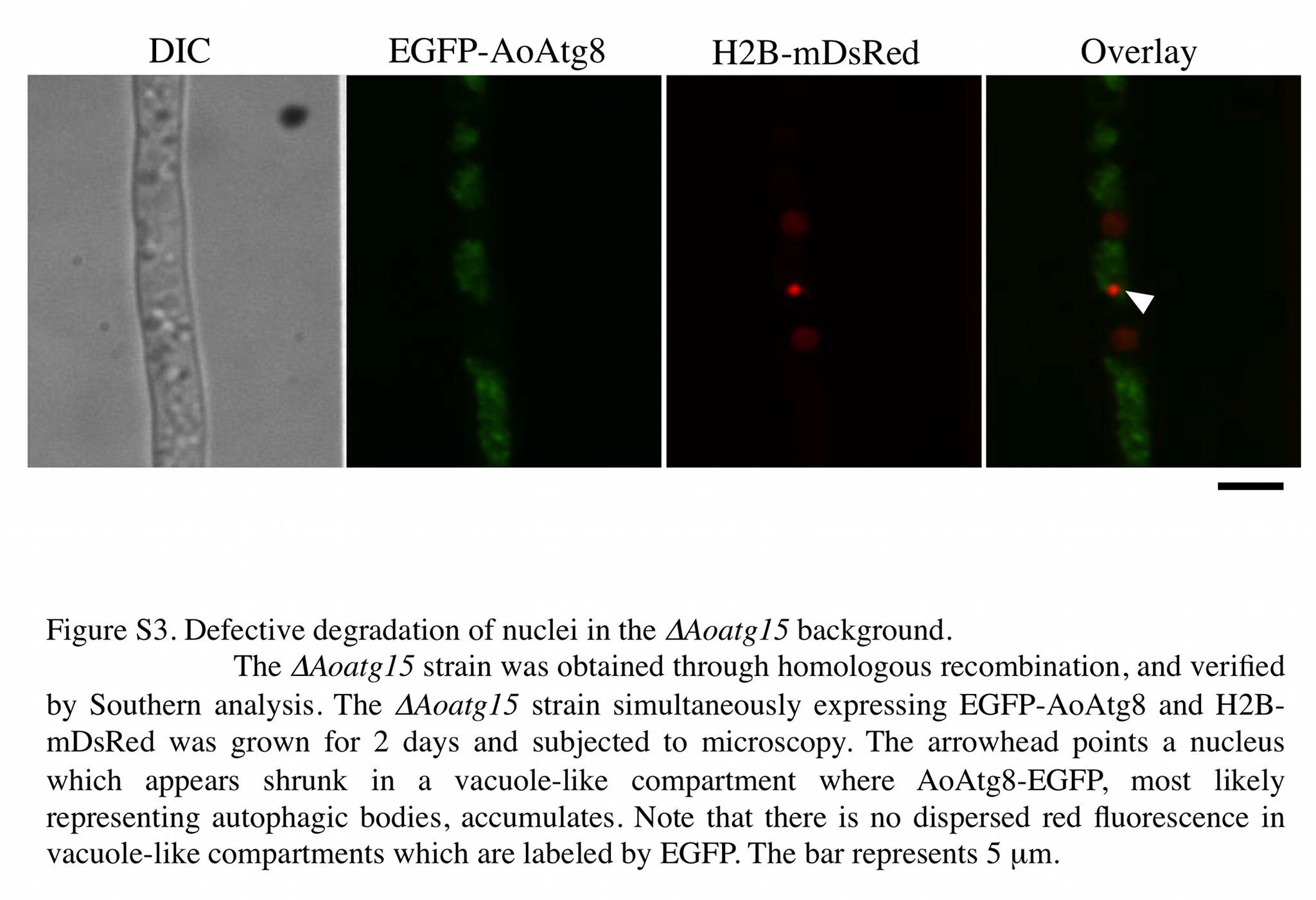

Supplement: Figure S3 — Defective degradation of nuclei in the ΔAoatg15 background. The ΔAoatg15 strain was obtained through homologous recombination, and verified by Southern analysis. The ΔAoatg15 strain simultaneously expressing EGFP-AoAtg8 and H2B-mDsRed was grown for 2 days and subjected to microscopy. The arrowhead points a nucleus which appears shrunk in a vacuole-like compartment where EGFP-AoAtg8, most likely representing autophagic bodies, accumulates. Note that there is no dispersed red fluorescence in vacuole-like compartments which are labeled by EGFP. The bar represents 5 µm. (TIF) [file pone.0015650.s004.tif]

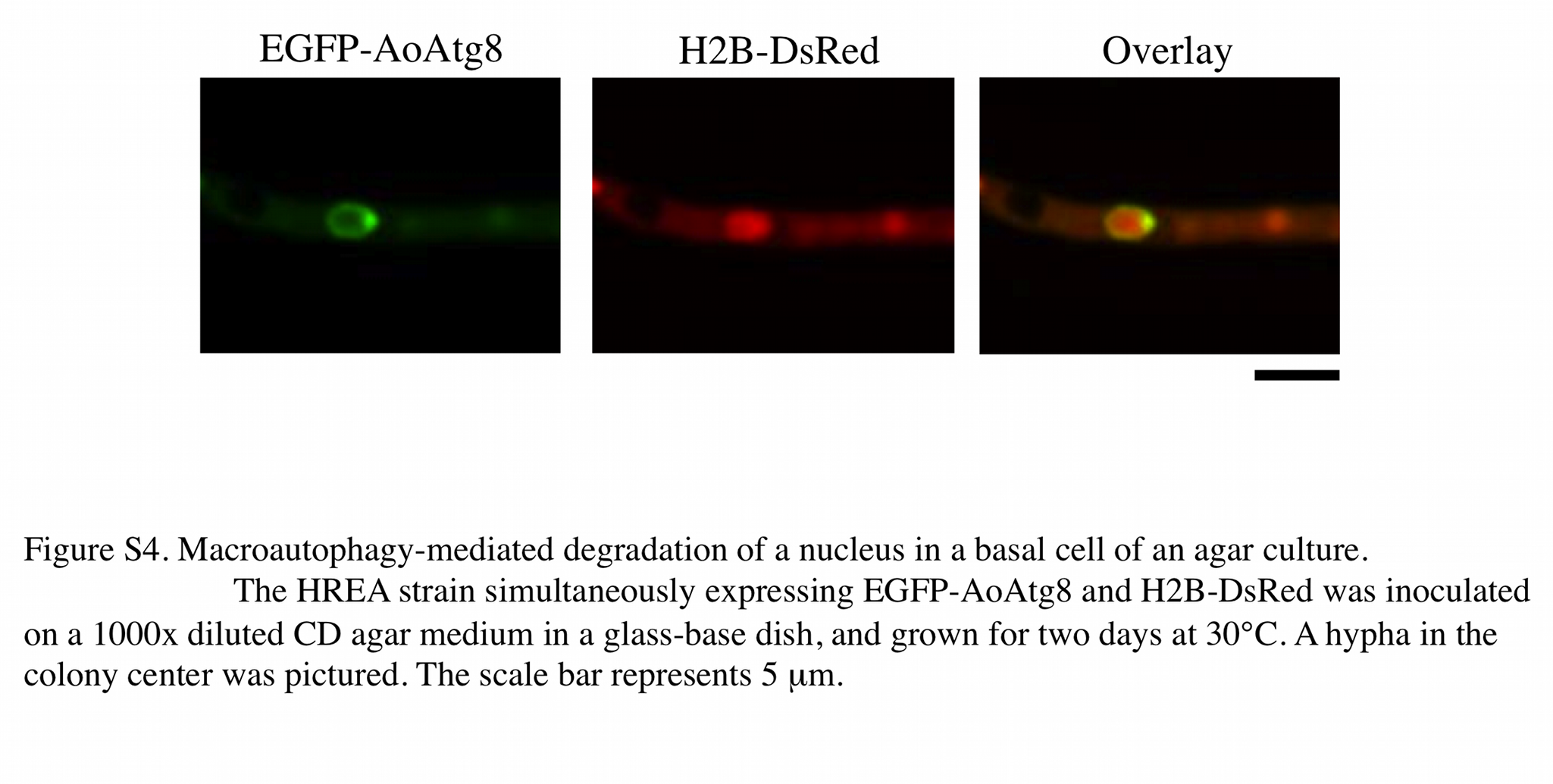

Supplement: Figure S4 — Macroautophagy-mediated degradation of a nucleus in a basal cell of an agar culture. The HREA strain simultaneously expressing EGFP-AoAtg8 and H2B-DsRed was inoculated on a 1000x diluted CD agar medium in a glass-base dish, and grown for two days at 30°C. A hypha in the colony center was pictured. The scale bar represents 5 µm. (TIF) [file pone.0015650.s005.tif]
